# Case report of multidisciplinary approach to post single anastomosis sleeve jejunal bypass (SASJ) surgery refusal to eat^☆^

**DOI:** 10.1016/j.ijscr.2022.107702

**Published:** 2022-09-27

**Authors:** Seyed Hadi Mirhashemi, Samaneh Jam, Samareh Omidvari, Yaser Samadi, Setareh Shishvan, Azadeh Hakakzadeh

**Affiliations:** a Department of the General Surgery, Loghman Hakim Hospital, Shahid Beheshti University of Medical Sciences, Tehran, Iran; b Clinical Research Development Unit of Loghman Hakim Hospital, Shahid Beheshti University of Medical Sciences, Tehran, Iran

**Keywords:** Obesity, Bariatric surgery, Laparoscopy, Psychology

## Abstract

**Introduction and importance:**

Bariatric surgeries are introduced as novel procedures in the whole world. Among the most important side effects after these surgeries is malnutrition. One of the reasons for suffocation can be the patient's psychological problems (such as depression). Paying attention to these symptoms can be effective in managing post-surgical complications.

**Case presentation:**

A 36-year-old female patient who was operated with SASJ BYPASS surgery method presented three weeks after the surgery with symptoms of weakness, lethargy, nausea, vomiting, and PO (Per OS) intolerance, which did not respond to outpatient treatment. Barium swallow imaging and abdominopelvic CT scan was done for the patient and findings were normal. During conservative treatment and total parenteral nutrition (TPN) the patient underwent psychiatric consultation and took psychiatry medications. Gradually after these consultation sessions the patient had a good PO tolerance, no edema and no weakness and was discharged in a good condition. She was advised to continue psychologic consultation sessions besides other post-surgical follow ups.

**Clinical discussion:**

After complete assessment of malnutrion etiologies after bariatric surgeries it was advised to ensure that the patients demonstrate an understanding of the bariatric surgical procedure, necessity of changes in eating habits. Any existing psychological issues should be identified and treated the patient should be educated to make a commitment to multidisciplinary care after these surgeries.

**Conclusion:**

With continued communication, support, and multidisciplinary monitoring, nutritional complications can be minimized among patients undergoing bariatric surgeries.

Level of evidence: V.

## Highlights

• Amongst the most important side effects after bariatric surgeries is malnutrition.
• One of the reasons for suffocation can be the patient's psychological problems (such as depression).
• With continued multidisciplinary monitoring, nutritional complications can be minimized among these patients.

## 1 Introduction

In the last 50 years, obesity has developed into a global public health problem that has an impact on people's quality of life, increasing their risk of comorbidities (cardiovascular disease, diabetes, and cancer) [1], and drives up medical expenses in nations all over the world. The body-mass index (BMI; weight in kg/height in m2), which has a high connection with body fat, is used in these surveys to measure obesity [2]. Obesity can be treated with lifestyle modifications (dietary restraints and increased physical activity), pharmaceutical usage, and in rare circumstances, surgery [3]. The most effective treatment in extreme obesity is bariatric surgery [4]. Bariatric surgery has grown in popularity as a therapeutic option for extreme obesity. In 2013, almost 500,000 operations were performed globally [2].

There are different methods for bariatric surgery procedures. The single-anastomosis sleeve jejunal (SASJ) bypass is a successful primary bariatric operation with various benefits, including simplicity and robustness, reduced malnutrition compared to other malabsorptive surgeries, and simple screening of the upper gastrointestinal tract and biliary tree [5]. Like other surgeries, bariatric surgeries have major and minor complications that can cause readmission to the hospital or reoperation. One of the most common complications is malnutrition after surgery. Bypassing the primary locations where micronutrient absorption takes place during malabsorptive operations is the main contributor to vitamin, mineral, and trace element deficiencies [6].

According to previous studies, a significant percentage of patients undergoing bariatric surgery also report changes in eating habits in addition to depressive and anxiety symptoms. However, because postoperative assessments were carried out at various intervals, they produced inconsistent outcomes. Studies evaluating depression, anxiety, and eating disorder symptoms one or two years following surgery reveal a notable reduction in symptoms [7]. Participants in the 2020 study on bariatric surgery patients felt that psychologists could help patients by addressing (after surgery) issues that may affect weight loss, such as reasons for obesity, relationships with food, and practical strategies for change. This would increase the likelihood that treatment would be successful. The majority of participants agreed that understanding each person's motivations for being fat was crucial to maintaining weight reduction [8]. Herein, we have reported a multidisciplinary approach to post-surgical refusal to eat, paying attention to psychiatric counseling and its treatment, and the importance of the permanent presence of a psychiatrist in the surgical team.

## 2 Case presentation

A 36-year-old woman, married and a mother of two kids, who suffered from morbid obesity, was a candidate for bariatric surgery. The presentation has been gathered according to the SCARE 2020 Guideline [9]. In her past medical history, she had a duodenal ulcer and hypothyroidism, and the hypothyroidism was treated with levothyroxine. She was mentioned an initial weight of 113 kg, BMI 41 kg/m^2^. The patient was a candidate for SASJ Bypass surgery due to her tendency to squint, high volume of food consumption, and affinity for sweets [10]. Three weeks after the surgery, the patient felt dyspepsia, nausea, and vomiting. The patient initially received oral treatment and partial recovery was achieved, but a few days later, the patient's symptoms (nausea and vomiting) got worse and became oral intolerance. The patient was also advised to consult her symptoms with psychologist but she refused it. Therefore, she became a candidate for hospitalization and further evaluation.

**Fig. 1:**
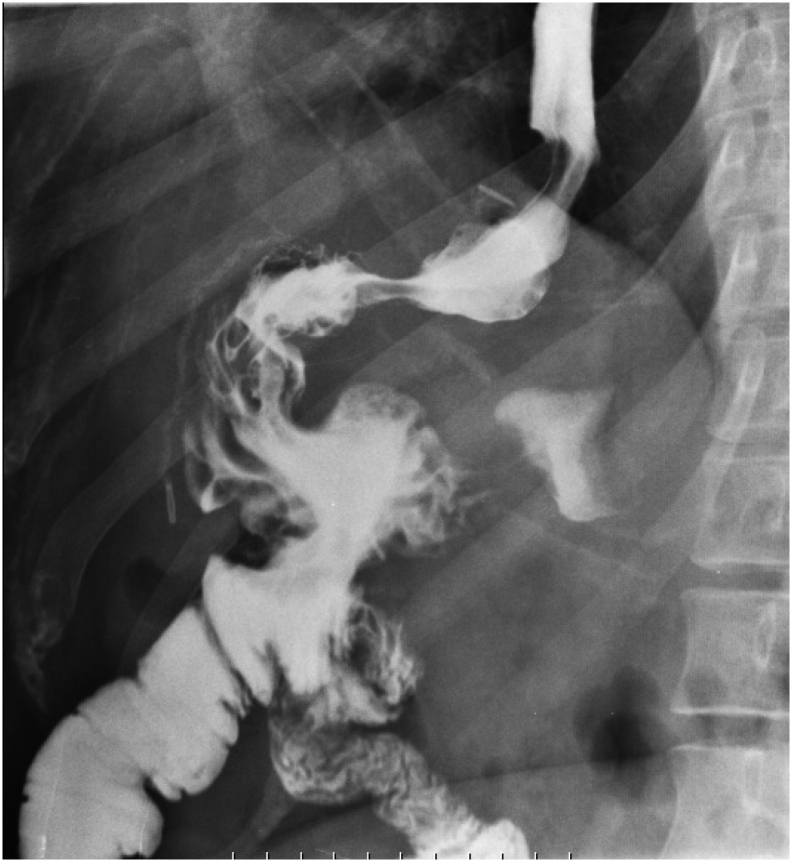
Barium swallow and meal radiography revealed normal gastroesophageal tract.

**Fig. 2:**
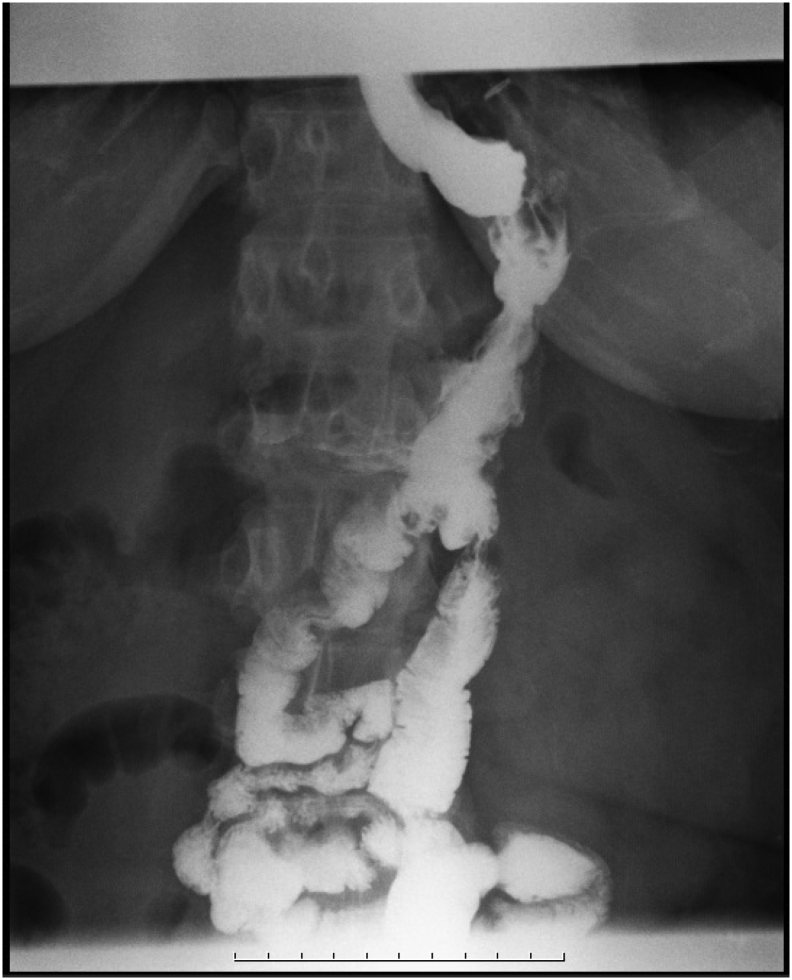
Barium swallow and meal radiography.

In the first hospitalization, upon admission in the general surgery department, the patient was evaluated clinically and paraclinically. Complementary laboratory tests were requested; Na (136 mg/dl), K (4.3 mmol/l), Urea (24 mg/dl), Cr (1.3 mg/dl), white blood cell count (4900 per microliter), Hb (12.8 mg/dl), PLT (210000), serum albumin and total protein, copper, calcium, phosphorus, 25 OH D3, serum iron, ferritin, liver function and thyroid function tests were all in normal range. In the report description of endoscopy bile reflux was found. Anastomosis and other findings were normal. Barium swallow imaging was done for the patient (Fig. 1, Fig. 2), and slow entry of swallowed substances from the esophagus into the stomach, reducing peristalsis, and delaying the exit of substances from the stomach were seen. Other findings were normal.

**Fig. 3:**
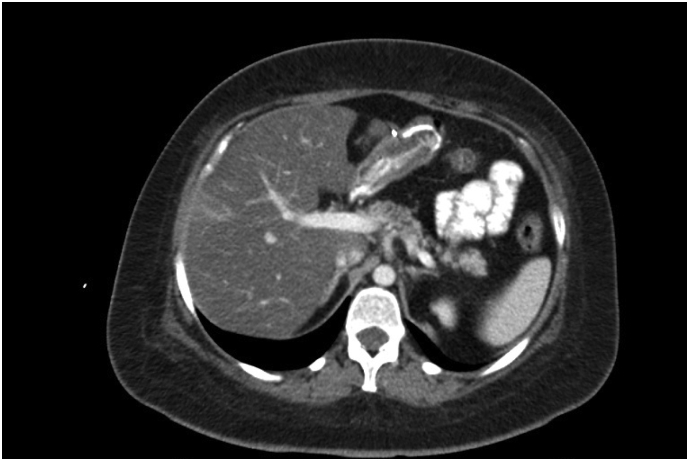
Abdominopelvic CT scan with intravenous and oral contrast agent.

**Fig. 4:**
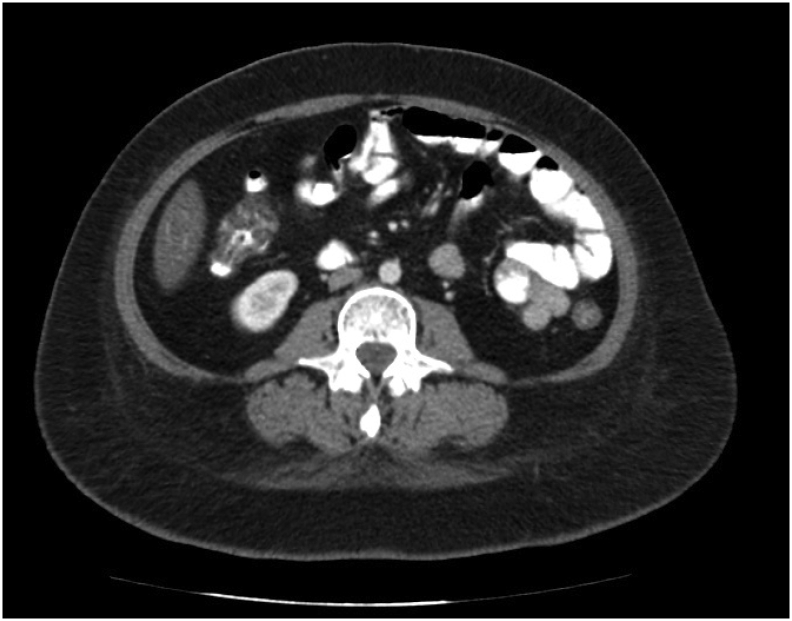
Abdominopelvic CT scan with intravenous and oral contrast agent.

In the abdominopelvic CT scan, contrast leak and other pathological findings were not reported. (Except a hyperdense liver lesion with the diagnostic possibility of sparing focal fat or perfusion change.) (Fig. 3, Fig. 4).

The patient underwent conservative treatment with appropriate serum therapy and injectable Ondansetron and Pantoprazole. Also, psychiatric consultation was requested. The patient was discharged from the hospital five days after being admitted because of a relative improvement in the patient's overall health, the acceptance of the laboratory and paraclinical results, and the lack of pathology necessitating further treatment. They also noted that the patient's general condition had improved somewhat and that there had been no nausea and vomiting. The patient's weight was 89 kg when discharged. At this stage, the patient didn't take psychological drugs and left the treatment team, and after the active follow-up of the patient was found by the treatment team, it was found that the patient was suffering from depression and had family problems.

Three months later the patient again suffered from nausea and vomiting, anasarca edema, and severe weakness and lethargy so that it became difficult for her to perform simple routine activities and even walk. Therefore, the patient was re-admitted and re-evaluated. During the second hospitalization **c**omplementary laboratory tests were requested; Aspartate aminotransferase(AST) (76 U/L), Alanine transaminase(ALT) (193 U/L), Bilirubin (T, D) (3.5/2 mg/dl), albumin (2.8 g/dl), total protein (4.3 g/dl), Zinc (91 μg/dl), Ferritin (194 ng/ml), VitB12 (1837 pg/ml), Folic acid 9.5 (nmol/L), VitD3 (109 ng/ml), and anemia (Hb 11.8 mg/dl) were detected. Liver Sonography was done. Fatty liver was seen; ascites was not found. Esophagus and Stomach manometry was done and revealed hypotensive lower esophageal sphincter pressure (8 mmHg) with hiatal hernia and ineffective esophageal motility indicating pseudoachalasia.

A CV line was inserted, and the patient was treated with total parenteral nutrition (TPN). Due to edema and a decrease in albumin, she was treated with injectable albumin and then oral protein supplements. The patient was given reassurance and also underwent psychiatric counseling again and this time the patient underwent psychotherapeutic treatment. She was advised to take fluoxetine 20 mg once a day and clonazepam 2 mg every night and to continue therapy sessions. Also, she was recommended to start aerobic exercise (walking) with moderate intensity 5 days a week.

After 16 days of hospitalization, and after correction of albumin and storage of vitamins, the patient had a good PO tolerance, without any edema, no weakness, or lethargy, with a good general condition and a weight of 85 kg. She was discharged with a high protein diet, pantoprazole tab, ondansetron tab, FEFOL tab, domperidone tab, fluoxetine and clonazepam. In the 6th month postsurgical follow-up visit, the patient was tolerating PO, she had no nausea and vomiting. No edema was found in physical assessment. The lab data was normal. She has lost weight of up to 77 kg and she was continuing her psychotherapy sessions and aerobic exercise.

## 3 Discussion

Many patients experience vomiting in the first 4 to 6 weeks after surgery, usually from eating too quickly, eating too much, eating foods not previously tolerated, or consuming excessive amounts of liquids during meals [11]. Most patients quickly learn how to avoid eating behavior that triggers vomiting. If vomiting persists after 6 weeks and no medical cause is found, the patients should be reevaluated for depression, post-traumatic stress disorders, relationship issues, and inadequate problem-solving skills. In this study we have presented a female patient with no previous history of psychological problems in preoperative surgical assessments who presented with dyspepsia, vomiting and refusal to eat after SASJ bypass surgery. Extreme obesity is associated with significant psychiatric co-morbidity [12]. Preoperative clinical interviews of patients have suggested that 20 % to 70 % suffer from a current or past psychiatric disorder. Mood disorders are the most common conditions, diagnosed in 19 %–60 % of patients [12]. It should be reemphasized that psychological evaluation and treatment of identified psychological and psychiatric disorders before bariatric surgery. Closed monitoring of behavioral symptoms after surgery and being aware of them including snacking, drinking soda, not taking vitamins and not exercising should also be concerned.

## 4 Conclusions

With continued communication, support, and multidisciplinary monitoring, nutritional complications can be minimized among patients undergoing bariatric surgeries. Given the increasing population of bariatric surgery patients, evaluation of patients' preoperative psychiatric status and routine psychological treatment sessions after surgery may play an important role in maximizing successful postoperative outcomes. It might be concerned that not only all surgeons should be aware of patients' physical and mental symptoms but also monitor their psychology consultations closely after bariatric surgeries.

## Provenance and peer review

Not commissioned, externally peer-reviewed.

## Consent

Written informed consent was obtained from the patient for publication of this case report and accompanying images. A copy of the written consent is available for review by the Editor-in Chief of this journal on request.

## Funding

There was no funding source for this study.

## Ethical approval

Not relevant.

## Author contribution

All authors made a major contribution to prepare the manuscript.

## Guarantor

Seyed Hadi Mirhashemi.

## Research registration

Not relevant.

## Declaration of competing interest

The authors declare no potential conflicts of interests with respect to the research, authorship, and/or publication of this article.
